# Supplementary material for: Clinical Activity of an hTERT-Specific Cancer Vaccine (Vx-001) in “Immune Desert” NSCLC
Source: Cancers (Basel). 2021 Apr 1;13(7):1658. doi: 10.3390/cancers13071658 (PMC8037524; doi:10.3390/cancers13071658)
Supplement: Supplementary file 1 [file cancers-13-01658-s001.pdf]

cancers

MDPI

Supplementary materials: Clinical Activity of an hTERT-Specific Cancer Vaccine (Vx-001) in "Immune Desert" NSCLC

I. S. Pateras, A. Kotsakis, M. Avgeris, E. Baliou, P. Kouroupakis, E. Patsea, V. Georgoulias, J. Menez-Jamet, J. P. Kinet and Kostas Kosmatopoulos

Table S1. Correlation of TAIC with CD3- and CD8- TILs.

	Tumor-associated immune cells (TAIC)		
**Covariant**	Low	High	*p*-value[a]
**CD3-TILs**			
Low	30[b] (68.2%[c])	15 (24.6%)	
High	14 (31.8%)	46 (75.4%)	<0.001
**CD8-TILs**			
Low	29 (65.9%)	13 (22.8%)	
High	15 (34.1%)	44 (77.2%)	<0.001

[a] Fisher's Exact test. [b] Number of patients. [c] % percentage within TAIC.

Table S2. Correlation of PD-L1, TAIC, CD3- TILs, CD8-TILs and GZMB-TILs with patients' clinicopathological data.

	**Gender**			**Age**			**Prior treatment response**			**Histology**			**Smoking**		
**Covariant**	Male	Female	*p*[a]	<65y	≥65y	*p*[a]	OR[d]	SD[e]	*p*[a]	NSQ/MH[f]	SQ[g]	*p*[a]	No	Yes	*p*[a]
**PD-L1**															
Negative	75[b] (53.7%[c])	26 (19.8%)	0.486	48 (36.6%)	53 (40.5%)	0.838	46 (35.1%)	55 (42.0%)	0.100	59 (45.0%)	42 (32.1%)	0.677	9 (6.9%)	92 (70.2%)	0.493
Positive	20 (15.3%)	10 (7.6%)		15 (11.5%)	15 (11.5%)		19 (14.5%)	11 (8.4%)		16 (12.2%)	14 (10.7%)		4 (3.1%)	26 (19.8%)	
**TAIC**															
Low	40 (30.5%)	20 (15.3%)	0.176	32 (24.4%)	28 (21.4%)	0.296	31 (23.7%)	29 (22.1%)	0.727	35 (26.7%)	25 (19.1%)	0.861	6 (4.6%)	54 (41.2%)	1.000
High	55 (42.0%)	16 (12.2%)		31 (23.7%)	40 (30.5%)		34 (26.0%)	37 (28.2%)		40 (30.5%)	31 (23.7%)		7 (5.3%)	64 (48.9%)	
**CD3-TILs**															
Low	35 (33.3%)	10 (9.5%)	0.508	22 (21.0%)	23 (21.9%)	1.000	23 (21.9%)	22 (21.0%)	0.846	27 (25.7%)	18 (17.1%)	0.554	5 (4.8%)	40 (38.1%)	1.000
High	43 (41.0%)	17 (16.2%)		30 (28.6%)	30 (28.6%)		32 (30.5%)	28 (26.7%)		32 (30.5%)	28 (26.7%)		6 (5.7%)	54 (51.4%)	
**CD8-TILs**															
Low	32 (31.7%)	10 (9.9%)	0.506	21 (20.8%)	21 (20.8%)	0.842	22 (21.8%)	20 (19.8%)	0.549	23 (22.8%)	19 (18.8%)	0.842	5 (5.0%)	37 (36.6%)	0.738
High	41 (40.6%)	18 (17.8%)		28 (27.7%)	31 (30.7%)		27 (26.7%)	32 (31.7%)		31 (30.7%)	28 (27.7%)		5 (5.0%)	54 (53.5%)	
**GZMB-TILs**															
Low	51 (60.0%)	19 (22.4%)	1.000	30 (35.3%)	40 (47.1%)	0.153	36 (42.4%)	34 (40.0%)	0.782	39 (45.9%)	31 (36.5%)	1.000	6 (7.1%)	64 (75.3%)	0.626
High	11 (12.9%)	4 (4.7%)		10 (11.8%)	5 (5.9%)		7 (8.2%)	8 (9.4%)		8 (9.4%)	7 (8.2%)		2 (2.4%)	13 (15.3%)	

[a] Fisher's Exact test, [b] Number of patients, [c] % percentage of total patients, [d] Optimal response, [e] Stable disease, [f] Non-squamous/mixed histology, [g] Squamous.

Table S3. Distribution of gender, age, response to previous treatment, tumor histology and smoking behaviour in Vx-001 and placebo-treated arms.

	Vaccination		
**Covariant**	Placebo	Vx-001	*p*-value[a]
**Gender**			
Male	51[b] (38.9%[c])	44 (33.6%)	
Female	17 (13.0%)	19 (14.5%)	0.560
**Age**			
<65y	32 (24.4%)	31 (23.7%)	
≥65y	36 (27.5%)	32 (24.4%)	0.862
**Response to previous treatment**			
OR[d]	38 (29.0%)	27 (20.6%)	0.163
SD[e]	30 (22.9%)	36 (27.5%)	
**Histology**			
NSQ/MH[f]	41 (31.3%)	34 (26.0%)	
SQ[g]	27 (20.6%)	29 (22.1%)	0.485
**Smoking**			
No	7 (5.3%)	6 (4.6%)	
Yes	61 (46.6%)	57 (43.5%)	1.000

[a] Fisher's Exact test, [b] Number of patients, [c] % percentage of total patients, [d] Optimal response, [e] Stable disease, [f] non-squamous/mixed histology, [g] squamous.

Table S4. OS in patients with PD-L1(+), TAIC high, CD3-TIL high, CD8-TIL high or GZMB-TIL high tumors.

Patients	OS (months)		HR[a]	95% CI[b]	log-rank p value
	Placebo	Vx-001			
PD-L1(+) (n=30)	24.9	7.9	1.646	0.647-4.186	0.291
TAIC high (n=71)	16.3	14.3	1.463	0.850-2.517	0.166
CD3-TIL high (n=60)	16.3	10.0	1.599	0.895-2.856	0.109
CD8-TIL high (n=59)	16.3	12.7	1.501	0.832-2.709	0.174
GZMB-TIL high (n=15)	21.0	4.6	3.751	1.022-13.77	0.034

[a] Hazard Ratio, [b] 95% confidence interval of the estimated HR.

Table S5. TTF in patients with PD-L1(+), TAIC high, CD3-TIL high, CD8-TIL high or GZMB-TIL high tumors.

Patients	TTF (months)		HR[a]	95% CI[b]	log-rank p value
	Placebo	Vx-001			
PD-L1(+) (n=30)	3.0	3.5	1.344	0.615-2.934	0.437
TAIC high (n=71)	2.8	2.3	1.040	0.639-1.693	0.869
CD3-TIL high (n=60)	3.0	2.3	1.201	0.701-2.058	0.484
CD8-TIL high (n=59)	3.0	2.3	1.208	0.705-2.068	0.473
GZMB-TIL high (n=15)	2.8	2.0	1.043	0.362-3.005	0.936

[a] Hazard Ratio, [b] 95% confidence interval of the estimated HR.

CD8/GZMB double staining

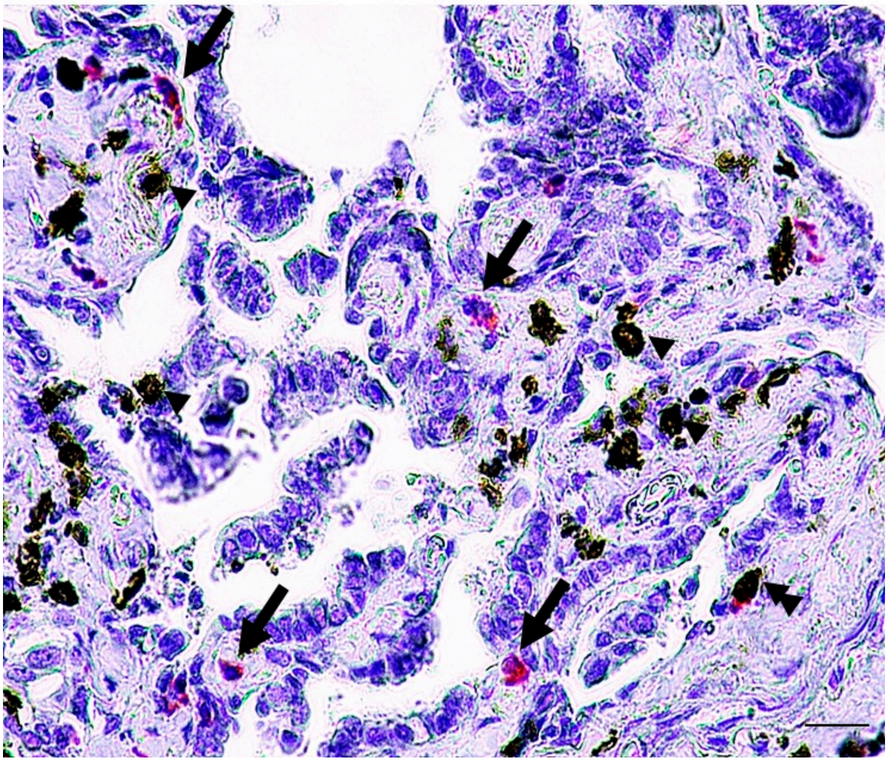

Figure S1. CD8-GZMB double staining. CD8 (brown, single arrowheads) – GZMB (red, single arrows). Double arrowhead depict CD8+GZMB+ cells. Scale bar: 20μm.

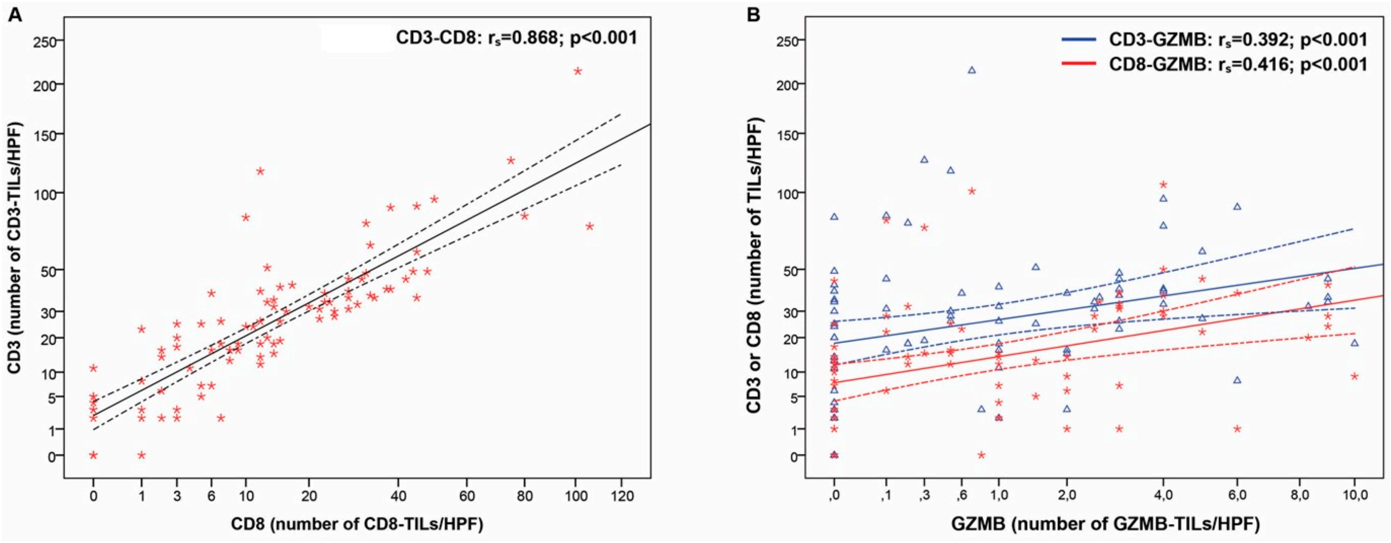

Figure S2. Correlation of CD3-TILs, CD8-TILs and GZMB-TILs. Scatter plots in power scale of (A) CD3-TILs and CD8-TILs and (B) GZMB-TILs with CD3-TILs (blue) or CD8-TILs (red). The *p* values are calculated by Spearman correlation analysis. The solid lines represent linear regression trendlines and dashed lines indicate 95% confidence intervals. r_s: Spearman correlation coefficient.

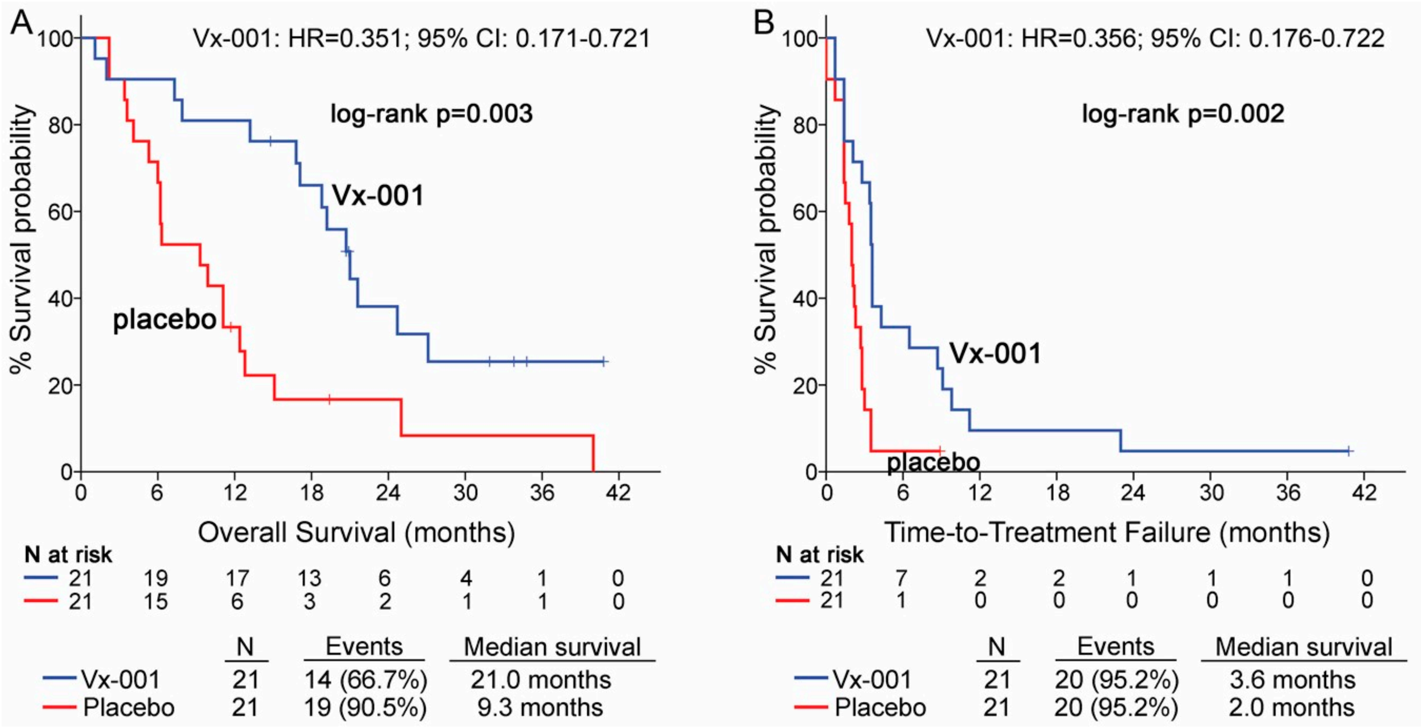

Figure S3. OS (A) and TTF (B) in patients with tumors lacking CD8-TIL/GZMB double positive cells.

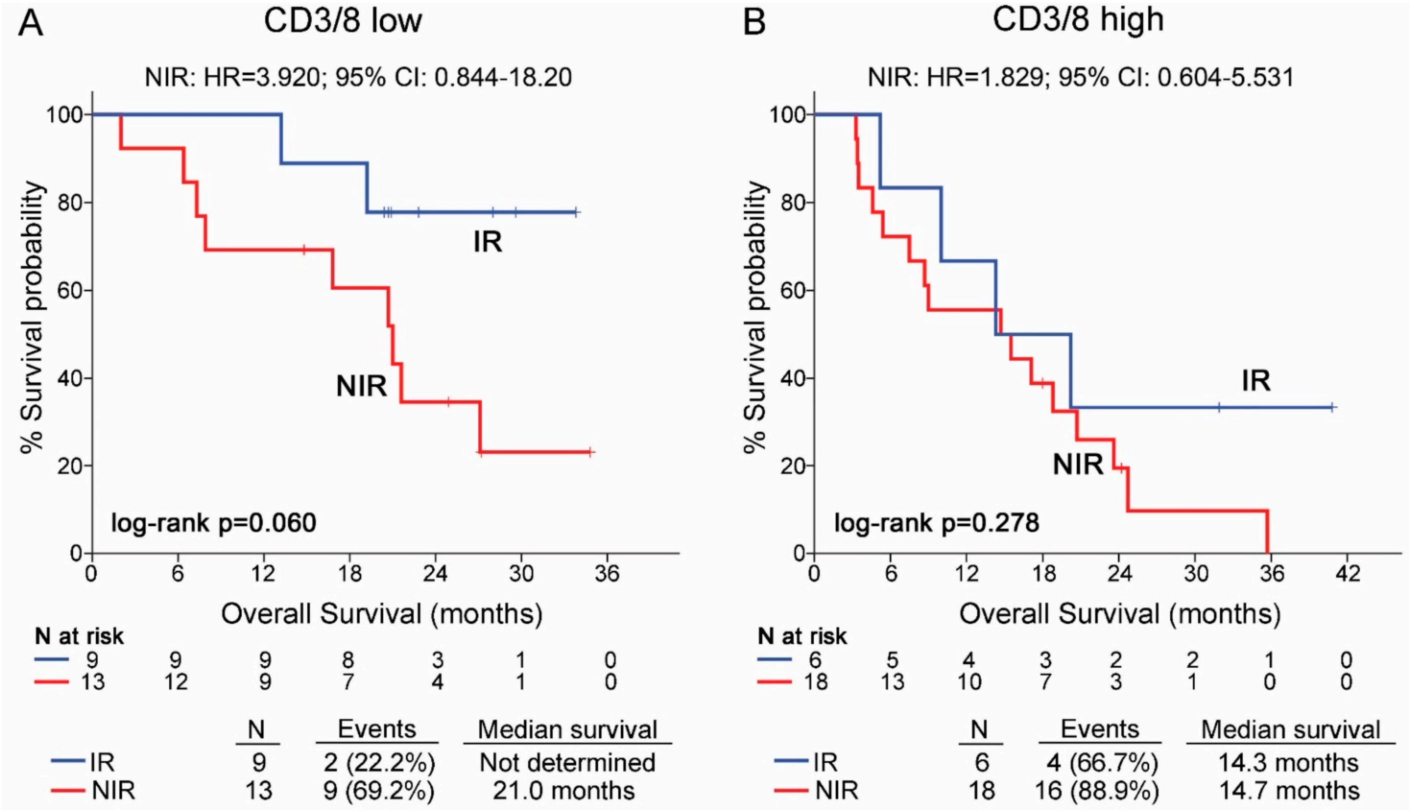

Figure S4. : Correlation of Vx-001 induced immune response and OS in patients with CD3/8-TIL low (A) and CD3/8-TIL high (B) tumors. IR: immune responders, NIR: Non immune responders.
